# Anethole Dithiolethione Increases Glutathione in Kidney by Inhibiting *γ*-Glutamyltranspeptidase: Biochemical Interpretation and Pharmacological Consequences

**DOI:** 10.1155/2020/3562972

**Published:** 2020-09-28

**Authors:** Daniela Giustarini, Federico Galvagni, Isabella Dalle-Donne, Aldo Milzani, Monica Lucattelli, Giovanna De Cunto, Desirée Bartolini, Francesco Galli, Annalisa Santucci, Ranieri Rossi

**Affiliations:** ^1^Department of Biotechnology, Chemistry and Pharmacy, University of Siena, Via A. Moro 2, I-53100 Siena, Italy; ^2^Department of Biosciences, University of Milan, Via Celoria 26, I-20133 Milan, Italy; ^3^Department of Molecular and Developmental Medicine, University of Siena, Via A. Moro 2, I-53100 Siena, Italy; ^4^Department of Pharmaceutical Sciences, University of Perugia, Italy

## Abstract

**Aims:**

Anethole dithiolethione (ADT) is a marketed drug to treat xerostomia. Its mechanism of action is still unknown, but several preclinical studies indicate that it is able to increase intracellular glutathione (GSH) and protect against oxidative stress. Here, we investigated the molecular mechanisms behind these effects.

**Results:**

Oral treatment of rats confirmed the GSH enhancing properties of ADT; among the different organs examined in this study, only the kidney showed a significant GSH increase that was already observed at low-dose treatments. The increase in GSH correlated with a decrease in *γ*-glutamyltranspeptidase (*γ*-GT) activity of the different tissues. *In vitro* and *ex vivo* experiments with tubular renal cells and isolated perfused rat kidney showed that the cellular uptake of intact GSH was correlated with the extracellular concentrations of GSH.

**Conclusion:**

s. The prominent *in vivo*pharmacological effect of ADT was a marked increase of GSH concentration in the kidney and a decrease of some systemic and renal biomarkers of oxidative stress. In particular, by inhibition of *γ*-GT activity, it decreased the production cysteinylglycine, a thiol that has prooxidant effects as the consequence of its autooxidation. The activity of ADT as GSH enhancer in both the circulation and the kidney was long-lasting. All these characteristics make ADT a promising drug to protect the kidney, and in particular proximal tubule cells, from xenobiotic-induced damage.

## 1. Introduction

Organosulfur compounds are organic compounds that contain one or more sulfur atoms, and they are often associated with intense foul odor. There are plenty of sulfur-containing molecules in the food chain, e.g., amino acids such as cysteine and methionine, lipoic acid (an essential cofactor of four mitochondrial enzyme complexes), allicin (the active flavor compound in crushed garlic), and bioactive compounds like dithiolethiones. The cyclic sulfur-containing molecules, called 3*H*-1,2-dithiole-3-thiones, are representatives of a specific class of sulfur-containing compounds found in trace amounts in certain cruciferous vegetables but also of synthetic origin. These agents are reported to possess antioxidant, chemoprotective, and chemotherapeutic activities [1]. Among these sulfur-containing compounds, a molecule of particular interest is anethole dithiolethione (ADT) [2]. Oltipraz is another organosulfur compound belonging to the dithiolethione class, which has been shown to have an antisteatotic effect in an *in vivo* trial in patients with nonalcoholic fatty liver disease [3].

Dithiolethiones have attracted scientific attention as potential cancer chemopreventive agents. Oltipraz has been reported to protect against carcinogenesis at various organ sites in animal models [4]. Oltipraz and ADT have also been evaluated in humans, but the available data did not provide convincing information on the efficacy of the treatments [2].

The chemoprotective effects of the organosulfur compounds may be related mainly to the increase in glutathione (GSH) and the phase II detoxification enzymes such as glutathione *S*-transferases (GST) [5]. GSH is the main cellular antioxidant and works by donating reducing equivalents and conjugating with electrophiles via GST catalysis in phase II detoxification reactions; therefore, its role is fundamental in the elimination of xenobiotics and reactive oxygen species (ROS) [6, 7]. It was also reported that ADT can activate Nrf2, which is a major transcriptional stimulator of antioxidant and cytoprotective genes and is critical for cancer prevention [8].

ADT is marketed in several countries as a choleretic and sialogogue drug, with a good record of safety and tolerability at the recommended therapeutic dose (~1 mg/kg/bw/day). It is known to increase hepatic GSH levels and to exert protection against the toxic effects of acetaminophen and carbon tetrachloride [9]. The exact mechanism through which ADT may increase GSH concentration is still far to be clarified. It was previously reported that the administration of ADT to mice elevated the activity of some enzymes involved in GSH production, such as glutamate-cysteine ligase and glutathione disulfide reductase (GR), glucose-6 phosphate dehydrogenase, and 6 phosphogluconic dehydrogenase, which replenish NADPH necessary for GR activity [5, 10]. However, we were not able to confirm these findings when ADT (or its main metabolite, ADTOH) was administered to rats [11, 12]. In the present investigation, we studied more in depth the effect of ADT on GSH levels by using rats as an animal model. The *in vivo* experiments confirmed that the GSH enhancing effect of ADT was maximal in the kidney and evidenced a significant decrease of *γ*-glutamyltranspeptidase activity. *In vitro* experiments were carried out both with cultured cells (HK-2) and the isolated perfused rat kidney in order to study the uptake of GSH into renal cells.

## 2. Materials and Methods

### 2.1. Materials

ADT was purchased from Hubei Zhonghecheng Chemical Co., Ltd. (Hubei, China); HPLC grade solvents were purchased from Mallinckrodt-Baker (Milan, Italy). All other reagents were obtained from Sigma-Aldrich, Milan, Italy, with the exception of some, indicated in the text.

Male Sprague-Dawley rats (300 g) were purchased from Charles River (Calco, Milan, Italy). Rats were kept under controlled conditions (22-24°C, relative humidity 40-50%, under a 12 h light/dark cycle) and fed ad libitum for 2-3 weeks before their use and during experiments. All animal manipulations were made in accordance with the European Community guidelines for the use of laboratory animals. The experiments were authorized by the local ethical committee of the University of Siena.

HK-2 (human kidney) cells, derived from normal proximal convolute tubule [13], were obtained from ATCCs (American Type Culture Collection Manassas, VA).

### 2.2. Rat Treatment and Sample Preparation for Analyses

Rats (*n* = 18) were orally administered 1 mg/kg (b.w.) ADT dissolved in saline (1 ml), once a day for three weeks or saline (control rats). At one-week intervals, 3 animals for both the ADT-treated group and control group were sacrificed for biochemical analyses. The animals were maintained into metabolic cages for urine collection. Blood was collected in EDTA-containing tubes from the abdominal aorta under pentobarbital anesthesia; organs were rapidly removed in the following order: liver, kidneys, lungs, heart, testis, and brain, washed in NaCl 0.9% (*w*/*v*), weighted, frozen in liquid nitrogen, and stored at -80°C until analysis. The right kidneys were excised and fixed with buffered formalin (10% *v*/*v*) for 24 h for the immunohistochemical analysis. The tissues were then dehydrated, cleared in toluene, embedded in paraffin, and cut into 6 *μ*m sections for immunohistochemical analysis.

Biochemical analyses were carried out in red blood cells (RBCs), plasma, tissue homogenates, and urine. One aliquot of blood (2 ml) was rapidly treated with 50 mM (final concentration) *N*-ethylmaleimide (NEM), and, after a 1 min incubation, plasma and RBCs were separated by 30 s centrifugation at 10,000 g. Plasma samples were used for low molecular mass disulfides (LMM-SS) and *S*-thiolated protein (RSSP) analyses. The plasma used for low molecular mass thiols (LMM-SH), protein thiols, and malondialdehyde (MDA) analysis was obtained by centrifugation of the remaining whole blood. Aliquots of plasma (0.2 ml) were immediately acidified by the addition of 5% (*w*/*v*, final concentration) trichloroacetic acid (TCA) containing 1 mM K_3_EDTA and used for LMM-SH analysis. Red blood cells were purified by repeated washings with phosphate-buffered saline solution (PBS) containing 5 mM NEM. Excised organs were homogenized in five volumes of ice-cold 20 mM Tris/Cl, pH 7.6, by a glass/teflon potter. Aliquots of samples (0.5 ml) were immediately acidified by the addition of 5 volumes of 8% (*w*/*v*) TCA containing 1 mM K_3_EDTA for LMM-SH analysis. 24 h urine was collected and stored at -80°C for total GSH analysis.

### 2.3. Analysis of Thiols and Disulfides in Blood, Selected Organs, and Urine

LMM-SH analysis in both deproteinized plasma and supernatants of homogenized organs was carried out by –SH group labeling with monobromobimane (mBrB) and separation by reversed-phase HPLC. GSH, cysteine (Cys), cysteinylglycine (CysGly), homocysteine (Hcys), and *γ*-glutamylcysteine (*γ*-GluCys) were separated and quantified by a single run [14]. Protein thiol levels in plasma were quantified by colorimetric reaction with 5,5′-dithiobis-(2-nitrobenzoic acid) (DTNB) by spectrophotometer [15]. Plasma LMM-SS and RSSP analyses were carried out on supernatants and protein pellets, respectively, obtained by acidification with 5% (*w*/*v*, final concentration) TCA of NEM pretreated samples. The thiols released after reduction with dithiothreitol (DTT) were quantified by HPLC following labelling with mBrB [14]. Protein thiolation index (PTI) was calculated as the molar ratio between RSSP (where RS is Cys, CysGly, Hcys, *γ*-GluCys, and GSH) and the concentration of free, DTNB-titrable protein thiol groups [16]. For analyses in RBCs, 0.15 ml aliquots of pelleted RBCs were 1 : 1 acidified with 15% (*w*/*v*) TCA. GSH and GSSG were measured in the supernatant, respectively, by analyzing the GS-NEM conjugate by HPLC and using the GSH recycling method at the spectrophotometer [17]. The rest of the packed erythrocytes were hemolysed by addition of 1 ml of 5 mM phosphate buffer, pH 6.5, containing 2 mM NEM, and centrifuged at 20,000 × g for 15 min at 4°C for the analyses of membrane skeletal proteins. The pellets were then resuspended with a glass rod in 5 mM phosphate buffer, pH 6.5, containing 1 mM NEM, and centrifuged at 20,000 × g for 15 min at 4°C; this step was repeated three times [18]. Urinary total glutathione (i.e., the sum of the reduced and the oxidized forms) was measured by the reduction of disulfides with DTT and –SH group labeling with mBrB [11].

### 2.4. Analysis of MDA in Plasma

MDA was determined by HPLC in the clear supernatants after their deproteinization with 10% (*w*/*v*) TCA containing 1 mM K_3_EDTA [19].

### 2.5. Immunohistochemistry

Tissue sections were pretreated with 3% hydrogen peroxide to inhibit the activity of the endogenous peroxidases. All the sections were incubated with 3% bovine serum albumin for 1 h at room temperature to block nonspecific antibody binding. They were then incubated overnight at 4°C with rabbit monoclonal anti-mouse to GSH (Virogen, MA USA), diluted 1 : 50. The section slices were rinsed and incubated with rabbit anti-mouse IgG-peroxidase, diluted 1 : 200 for 30 min at room temperature. Color development was performed using 3,3′-diaminobenzidine tetrahydrochloride as a chromogen. As a negative control for the immunostaining, the primary antibody was replaced by no immunized serum.

### 2.6. Enzyme Activity

Enzyme assays were performed in homogenized rat organs. All enzymatic activities (except *γ*-GT activity) were determined on the supernatants obtained by the ultracentrifugation of homogenates (105,000 g for 1 h at 4°C). Glutathione S-transferase activity was determined by colorimetric analysis of the GSH-DNB conjugate at 340 nm [20]. Glutathione reductase activity was determined by colorimetric analysis of NADPH oxidation [21]. Catalase and glucose-6-phosphate dehydrogenase activities were assessed by measuring the hydrogen peroxide dismutation and NADPH production, respectively [22, 23]. Glutathione peroxidase activity was determined by the colorimetric analysis of NADPH oxidation coupled to GSH oxidation by cumene hydroperoxide [24]. Superoxide dismutase and *γ*-glutamyltranspeptidase (*γ*-GT) activity analyses were carried out by using pyrogallol and *γ*-glutamyl-3-carboxy-4-nitroanilide (*γ*-GCNA) as substrate, respectively [25, 26]. In those experiments where GSH was used as a substrate instead of *γ*-GCNA, the activity was measured by determining the formation of cysteine [25]. Glutamate-cysteine ligase and glutathione synthase activities were determined by measuring the production of *γ*-GluCys with time by HPLC [27] and ADP by spectrophotometer [28].

### 2.7. Analysis of Creatinine in 24 h Urine Samples

Analysis of creatinine was carried out on urine samples stored at -20°C according to Jaffe reaction [29]. Briefly, diluted samples (1 : 20) were reacted with picric acid under alkaline conditions to form a characteristic yellow-orange complex. After a 15 min incubation, the absorbance spectrum was analyzed in the wavelength range of 700-350 nm, revealing a peak at 490 nm. In order to remove from calculation the interference of nonspecific substances, the difference in color intensity measured at 490 nm before and after sample acidification was considered.

### 2.8. Cells

HK-2 cells were grown in Keratinocyte Serum-Free Medium (K-SFM, Invitrogen Life Science Technologies, Grand Island, NY, USA). Confluent cells were treated for 8 or 24 hours with 50 *μ*M ADT or vehicle alone (90% *v*/*v* ethanol +10% *v*/*v* dimethyl sulfoxide) in the presence or not of 50 *μ*M GSH. At the end of the treatment, the cells were washed twice with ice-cold PBS and lysed in 4% (*w*/*v*) TCA containing 1 mM K_3_EDTA.

### 2.9. Isolated Perfused Kidney

Experiments in the isolated perfused kidney (IPK) were carried out in the single-pass mode following an established procedure [30]. After clarification of the effluent, inulin (60 mg/100 ml) was added to the perfusion medium, and, after a 5 min equilibration period, the single-pass perfusion was started with the perfusate consisting of fraction V bovine serum albumin (BSA, 65 g/L), D-glucose (5 mM), L-cysteine (0.2 mM), glycine (2.0 mM), L-glutamic acid (0.5 mM) added with 5 *μ*M GSH (*n* = 3), 10 *μ*M GSH (*n* = 3), 20 *μ*M GSH (*n* = 3), 20 *μ*M GSH + 0.5 *μ*M ADT, 20 *μΜ*GSH + 0.1 mM probenecid (*n* = 3), 20 *μΜ*GSH + 1 mM BSO (*n* = 3). In one series of experiments (*n* = 3), the perfusion with 20 *μΜ* GSH was preceded by the perfusion with 0.5 mM acivicin (20 ml/min for 5 minutes, recirculating mode). The perfusion lasted 2 hours. Urine samples and perfused kidneys were stored at -20°C until analytical determinations. Samples were collected at 30, 60, and 90 minutes from the beginning of the perfusion from the reservoir connected to the renal artery and from the renal vein outflow for GSH analyses and calculation of its extraction rate. The functional viability of the kidney was assessed by measurement of the glomerular filtration rate (taken to be renal clearance of inulin) and the percentage of tubular reabsorption of electrolytes and glucose.

All the HPLC analyses were carried out by using a Zorbax Eclipse XDBC18 column (4.6 × 150 mm, 5 *μ*m, Agilent Technologies, Milan, Italy).

### 2.10. Protein Concentration Determination

Protein concentration was determined by the Bradford assay [31] using bovine serum albumin as a standard. Hemoglobin concentration was determined by analyzing the hemolysed RBCs by spectrophotometry in the 500-700 wavelength range and considering the peak height at 541 nm (*ε* = 13.8 mM^−1^ cm^−1^) [32].

### 2.11. Statistics

Data are the mean ± SD. Differences between means were evaluated by using ANOVA followed by Bonferroni posttest. A value of *p* < 0.05 was considered statistically significant.

## 3. Results

### 3.1. Levels of Thiol Redox Forms in Rat Organs, Blood, and Urine

**Figure 1:**
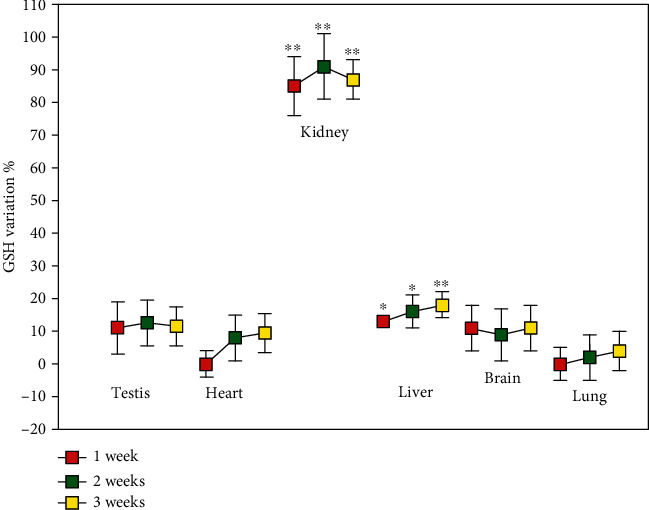
GSH levels in rat organs. Rats were orally administered 1 mg/kg/day ADT or vehicle. At 1-week intervals, some animals were sacrificed and the levels of GSH were measured in the indicated solid organs. Data are the mean ± SD (*n* = 3). ^∗^*p* < 0.05 vs. vehicle; ^∗∗^*p* < 0.01 vs. vehicle.

**Figure 2:**
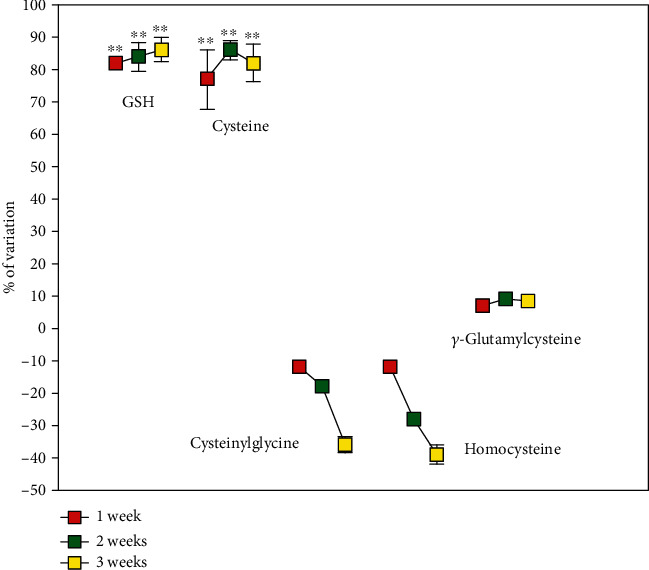
Low molecular mass thiol levels in rat kidney. Rats were orally administered 1 mg/kg/day ADT or vehicle. At 1-week intervals, some animals were sacrificed and the level of low molecular mass thiols was measured in the kidneys. Data are the mean ± SD (*n* = 3) ^∗∗^*p* < 0.001 vs. vehicle.

Figure 1 shows the levels of GSH in several rat organs at 1-week intervals during ATD treatment. After 1 week in vehicle-treated animals 2135 ± 119 nmoles of GSH/g of tissue were observed in the kidney, 4130 ± 3 60 nmoles/g in the testis, 1365 ± 210 nmoles/g in the heart, 7490 ± 560 nmoles/g in the liver, 1680 ± 105 nmoles/g in the brain, and 2219 ± 91 nmoles/g in the lung. The values were not significantly different after two and three weeks of saline administration. After a week of treatment with ATD, the kidney showed the highest increase in GSH (about 80% compared to vehicle), while in the other organs the GSH concentrations were not significantly increased, with the exception of the liver where the increase in GSH was significant compared to vehicle even if much smaller if compared with that of the kidney (18%, *p* < 0.01). The concentration of renal GSH remained almost doubled throughout the experiment, whereas in the other organs no increase in GSH was observed. The analysis of the other physiological low molecular mass thiols (LMM-SH) occurring in the kidney indicated that Hcys and CysGly decreased over the entire span of the treatment with ATD, while Cys increased along with GSH, from the first week of treatment. The levels of *γ*-GluCys were unvaried (Figure 2). After one week in vehicle-treated animals the levels of LMM-SH were: Cys =364 ± 73 nmoles/g tissue, *γ*-GluCys =9.10 ± 0.05 nmoles/g tissue, CysGly =3.55 ± 0.83 nmoles/g tissue and Hcys =4.97 ± 0.06 nmoles/g tissue. The values were not significantly different after two and three weeks of saline administration.

**Figure 3:**
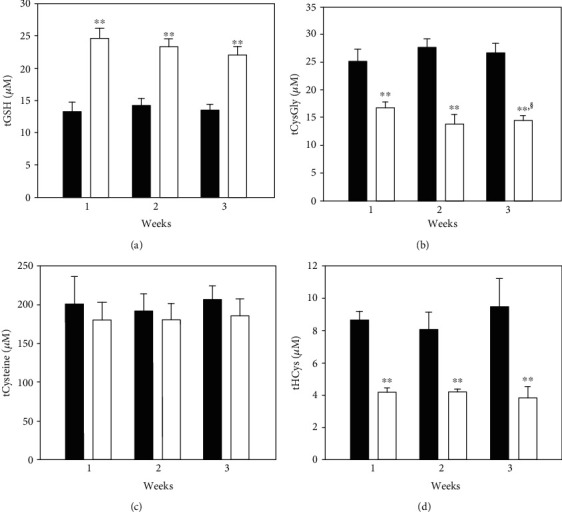
Total thiols in plasma. Rats were orally administered 1 mg/kg/day ADT (white columns) or vehicle (dark columns). At 1-week intervals, some animals were sacrificed and the levels of low molecular mass thiols (LMM-SH), low molecular mass disulfides (LMM-SS), and *S*-thiolated proteins (RSSP) for each thiol were measured in plasma. The total thiol levels (i.e., the sum of LMM − SH + 2 x LMM − SS + RSSP) are shown. Data are the mean of 3 experiments. ^∗∗^*p* < 0.01 vs. vehicle; ^§^*p* < 0.05 vs. 1-week ADT.

Treatment with ADT increased plasma tGSH after one week and this effectlasted throughout the other time-points of the treatment (*p* < 0.001 vs. vehicle, Figure 3(a)).tCys Gly and tHcys decreased. Specifically, both the reduced and disulfide forms of CysGly decreased (both as low molecular mass disulfide and as disulfide with proteins at most of the analyzed times, Table 1). The *S*-cysteinilated proteins and both the oxidized forms of homocysteine were also found to significantly decrease throughout the treatment span (Table 1). In close correlation with these alterations, several blood biomarkers of oxidative stress decreased, i.e., MDA and PTI in the plasma, and the concentration of membrane *S*-thiolated proteins in RBCs. Moreover, the GSH/GSSG ratio decrease in ADT-treated rats (Table 2).

**Figure 4:**
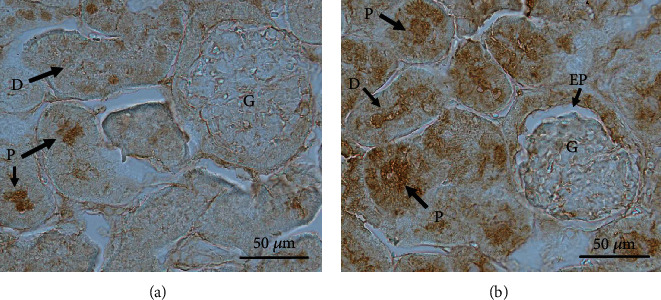
Representative immunohistochemical detection of GSH in rat kidney. Rats were orally administered 1 mg/kg/day ADT or vehicle. After 3-weeks treatment, the right kidney was excised for immunohistochemical evaluation. Constitutive distribution of GSH can be appreciated in proximal (P) and distal (D) tubules (arrows) in tissue sections from control rats (a). Only a faint reaction for GSH can be found in glomeruli from control rats treated with the solvent. A more intense and diffuse positive reaction is evident in proximal (P) and distal (D) tubules (arrows) from kidneys of GSH-treated rats (b). The immune reaction can be also appreciated in epithelial parietal cells of Bowman's capsule (EP) in kidney sections from treated rats.Bar = 50 *μ*m.

The cellular effects of ADT on the GSH levels in the kidneys were further verified by immunohistochemistry. As shown in Figure 4(b), GSH was mainly localized in the proximal and distal tubules of the renal cortex of ADT-treated rats. In some glomeruli of rats treated with ADT, GSH was identified in the epithelial cell layer of the Bowman's capsule (Figure 4(b), arrow EP). The distribution of GSH in the kidneys of control rats is revealed as a faint immune reaction in proximal and distal tubules (Figure 4(a)). No appreciable differences were observed in the intensity and distribution of the immunohistochemical reaction to GSH in renal medulla from both experimental groups (data not shown).

### 3.2. Enzyme Activities in Rat Organs

The reasons for the observed variations in GSH levels of the different organs (in particular in the kidneys) were sought by measuring their GSH-related enzymatic patterns. We did not find any change in the activity of the two enzymes involved in the synthesis of GSH, i.e., glutamate-cysteine ligase and GSH synthase. These data disagree with what is previously reported in the literature, where a significant increase in the activity of glutamate-cysteine ligase had been found in micetreated with ADT [10]. Analogously, all other enzymes that utilize GSH as a cofactor in conjugation/oxidation reactions (e.g., glutathione transferase and glutathione peroxidase),maintain its reduced form (e.g., glutathione reductase and glucose-6-phosphate dehydrogenase) or participate in ROS detoxification (catalase and superoxide dismutase), did not change their activity after treatment (data not shown). The only enzyme affected was *γ*-GT, whose activity resulted to be reduced in most of the analyzed organs, chiefly in the kidneys (about 30% decrease after 1-week treatment, Table 3).

The activity of *γ*-GT is generally measured by using the chromogenic substrate *γ*-GCNA [25]. However, a class of *γ*-GT-related enzymes with different *γ*-glutamylic-cleaving activity has been identified [33]. Therefore, we checked the inhibition of *γ*-GT using its natural substrate (i.e., GSH) instead of *γ*-GCNA. In the presence of GSH, the decrease in activity was even greater (around 50% at 50 *μ*M ADT concentration, data not shown). The decreased activity of *γ*-GT that we observed is likely not due to a direct inhibition of the enzyme by ADT. In fact, in subsequent experiments where a crude extract of rat kidney was treated with up to a final concentration of 1 mM ADT, we did not find any modification of *γ*-GT activity in the presence of both *γ*-GCNA and GSH as substrates (data not shown).

### 3.3. Cell Treatment

**Figure 5:**
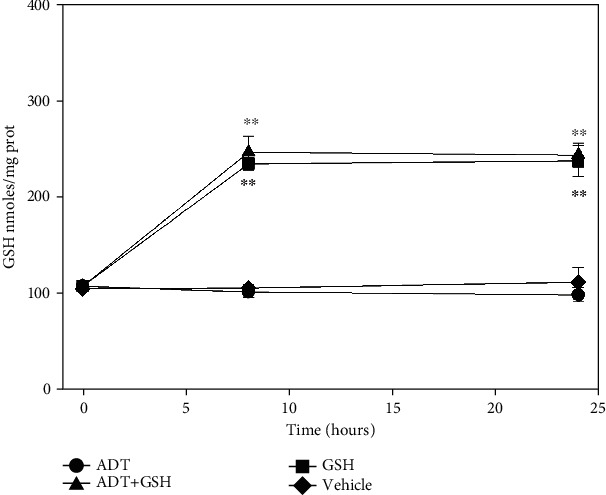
Levels of GSH in HK-2 cells. Cells were treated with GSH (squares), with vehicle (diamonds), or with 0.05 mM ADT, in the presence (triangles) or not (circles) of GSH. After 8 h and 24 h of treatment, the levels of GSH were measured. *n* = 3, ^∗∗^*p* < 0.01 vs. cells not treated with GSH.

The cellular pathways modified by ADT treatment were more thoroughly studied by using kidney tubular cells (HK-2 cells). However, *in vitro* treatments with ADT did not increase intracellular levels of GSH and/or its precursors/metabolites. Instead, in the presence of extracellular GSH, its concentration inside the cells almost doubled regardless of the presence of ADT (Figure 5). This result suggests that intact GSH could be taken by renal proximal tubular cells.

### 3.4. Isolated Perfused Rat Kidney

**Figure 6:**
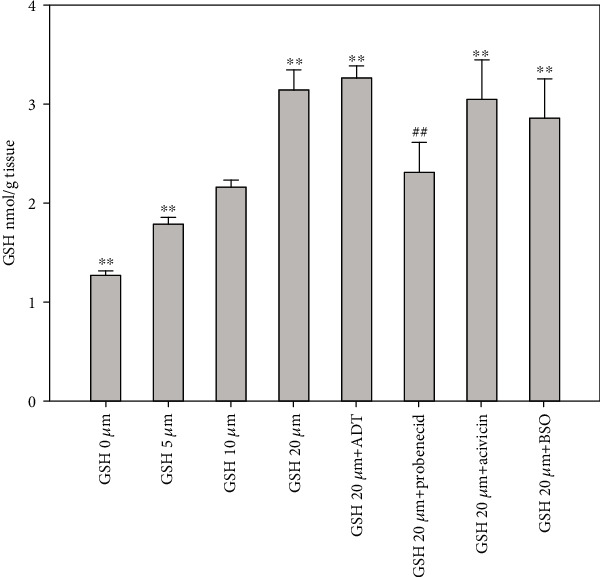
Levels of GSH in isolated perfused rat kidney. Rat kidneys were perfused for 2 hours with a standard physiological solution containing GSH at different concentrations or combinations of GSH with acivicin, probenecid, or BSO. At the end of the perfusion, the levels of GSH were measured by HPLC in kidney homogenates. *n* = 3, ^∗∗^*p* < 0.01 vs. GSH 10 *μ*M, ^##^*p* < 0.01 vs. GSH 20 *μ*M.

In order to study the renal metabolism of GSH, we used the isolated perfused rat kidney model. Kidneys were perfused with a solution resembling the composition of blood plasma as for the concentrations of proteins, amino acids and GSH. In this experiment, we challenged the kidneys with various concentrations of GSH (0, 5, 10, and 20 *μ*M). As shown in Figure 6, the levels of GSH in the kidneys increased proportionally to the increasing levels of GSH in the perfusion fluid. Concomitant treatments with acivicin and buthionine sulfoximine (BSO, an inhibitor of GSH synthesis) did not influence the renal levels of GSH. Instead, when probenecid was administered together with 20 *μ*M GSH, we observed a decrease in GSH level in the kidneys. These data indicate the presence of a peculiar transport system for GSH, independent from its degradation by *γ*-GT and suggest that most of the renal GSH depends on its influx from plasma rather than on its synthesis. Measuring the concentration of GSH in the artery and renal vein during the abovedescribed experiment, we calculated the extraction rate of the kidneys. The concentration of GSH sharply decreased after passing through the kidneys, and around 70% was removed at all the analyzed times also when the concentration of external GSH was twice the normal (i.e., 20 *μ*M vs. 10 *μ*M). The addition of acivicin to the perfusion medium evoked glutathionuria, but the removal rate of GSH did not change. Differently, probenecid decreased the overall renal removal of GSH without any evidence of glutathionuria (Table 4).

## 4. Discussion

The results presented in this study demonstrate that ADT acts as a GSH enhancer and that this effect is specific for the kidney tissue. Our data also suggest that the increase in GSH is presumably due to the enhanced direct influx of GSH into renal cells and to *γ*-GT inhibition. ADT is a marketed drug in several countries mainly used in Sjogren syndrome and symptomatic treatment of radiation-induced xerostomia in head and neck cancer patients (trade names: Sulfarlem S25, Sialor) [34, 35]. Its mechanism of actions is largely unknown, but it has been reported to have both direct and indirect antioxidant functions [1]. The notion that ADT is able to increase GSH levels in some target organs is not new, although in almost all previous experiments [9, 10, 12, 36] ADT was administered to experimental animals at very high doses for short periods. For example, in some preclinical studies, mice were treated by gastric intubation in the dose-range 10-125 mg/kg for 4 or 18 hours [36] or by gavage at 4.5 mmoles/kg for 48 or 96 hours [10]. Differently, in this study, we treated rats with a dose of ADT more comparable with that used for humans (~1 mg/kg/day) by performing long-term administration. This ruled out any possible direct antioxidant effect of ADT, since its maximal concentration reached during treatments was in the submicromolar range (not shown). Under these experimental conditions, the organ-specific activity of this drug was particularly evident, since GSH increased dramatically only in the kidneys and, to a minimal extent, in the liver (Figure 1). Among plasma LMM-SH, total GSH showed the most significant variation by doubling its concentration, whereas the concentrations of CysGly and Hcys (Figure 3) decreased in all the three redox forms, and cysteine decreased in the protein-bound form (CySSP, Table 1). The decrease of CysGly may play an important role for the mechanism of action of ADT. In fact, CysGly is very reactive and forms discrete amounts of peroxides [37, 38]; therefore, it is supposed to have prooxidant effects. The remarkable decrease in CysGly concentration is likely due to the antioxidant effect of ADT, which is demonstrated by several parameters of oxidative stress. These include PTI, an overall indicator of the disulfides to thiols ratio in plasma (Table 2), and total Hcys levels (50% of decrease after one week of treatment with ADT). Usually, most of the circulating Hcys is under the form of mixed disulfide with albumin [39], thus not available for cellular metabolism or renal excretion. The ADT-induced shift towards more reduced forms of this amino acid may lead its entry into cells for metabolization and/or renal clearance. The inhibition of *γ*-GT activity observed after ADT treatment may play a key role in generating all these events. Decreasing the decomposition of GSH into CysGly, may lead to a lower flux ROS thus reducing the levels of oxidative stress. *γ*-GT participates in the *γ*-glutamyl cycle by catalyzing the breakdown of extracellular GSH, generating CysGly and glutamic acid. CysGly is then hydrolyzed by a dipeptidase to Cys and glycine [40]. *γ*-GT is particularly present in the brush-border membrane (BBM) of kidney proximal tubule (PT) cells, where its main function is to cleave the GSH present in the glomerular filtration water. In fact, it is well known that treatments with the potent inhibitor of *γ*-GT acivicin evoke glutathionuria [41]. Furthermore, the kidney has a peculiar content of LMM-SH compared to other organs [42], particulalry Cys, which may derive from the GT-dependent hydrolysis of the GSH in the filtrate. Additionally, Cys can derive from glutathione *S*-conjugates that in the kidneys are transformed in the corresponding *S*-cysteine conjugates after their hydrolysis catalyzed by *γ*-GT and dipeptidase. *S*-cysteine conjugates are readsorbed by the active amino acid transporters of the kidney. Cys can be finally regenerated through the action of cysteine *β*-lyase [43]. The selective nephrotoxicity of a range of haloalkenes is attributable exactly to this bioactivation process that will be investigated in a further study. However, it could be of interest of the fact that we did not observe any significant inhibition of *γ*-GT in the experiments carried out with cultured cells (HK-2 cells) or with the IPK model.

It is also worth noting that ADT did not elicit any increase of cellular GSH when administered to HK-2 cells. Conversely, when GSH was added to the culture medium (which is generally devoid of GSH), a significant increase of this tripeptide was observed at the cellular level (Figure 5). The immunohistochemical analysis showed that after *in vivo* treatments with ADT, GSH mainly localizes in the proximal and distal tubules of the renal cortex (Figure 4(b)). This is in accordance with the*in vitro* data of Lash et al. [44]that described the presence of two transporters for intact GSH in these renal cells. This seems to be a peculiar property of the kidney, whereas it is widely debated that GSH can enter other mammalian cells. Most of tissues are considered impermeable to GSH with the exception of the intestine and brain [45].

**Figure 7:**
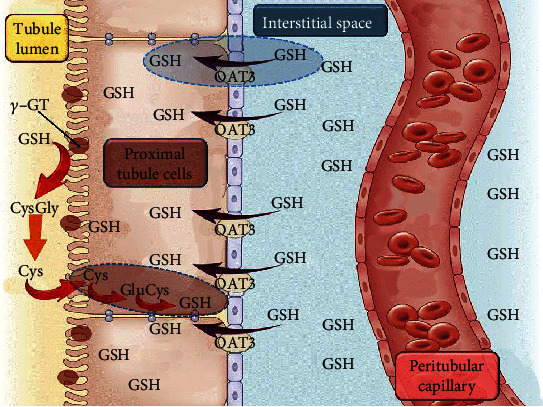
Schematic diagram representing the flux of GSH into the renal cells. GSH can enter renal cells by different pathways. Plasma GSH enters renal proximal tubular cells by specific carriers, Oat3 being one of the most representative. The lumen side of the tubular cells is rich in *γ*-GT that cleaves GSH into CysGly and glutamate. CysGly can be then degraded to the two constitutive amino acids by a dipeptidase. The cysteine (and other amino acids) can be reabsorbed into lumen tubular cells by specific transporters and GSH de novo resynthesized. The two pathways concur to maintain cellular levels of GSH.

The *ex vivo* experiments carried out by the IPK model confirmed that the levels of GSH in the kidney are controlled by external GSH, but are independent of *γ*-GT degradation (and successive Cys intake) at the PT level. The explanation of this could be found in the extensive and elegant study of Lash et al. [46], where they demonstrated that intact GSH can be transported into the cellular componets of the PT and (to a lesser extent) of the distal tubular (DT) cells, by means of Na^+^- and energy-dependent transporters. These transporters, located in the basolateral membrane and not in the BBM, have different kinetic parameters in PT and DT cells. Additionally, two populations of cells have been described in the PT, which show different affinity for GSH (*K*_*m*_ = 41.7 *μ*M and 540 *μ*M, *V*_max_ = 183 pmol/min × 10^6^ cells and 4885 pmol/min × 10^6^ cells, respectively). DT cells were shown to be less efficient than PT cells for GSH transport (*K*_*m*_ = 1480 *μ*M, *V*_max_ = 2984 pmol/min × 10^6^ cells). The cellular localization and the kinetic properties of these transporters can explain the results obtained in the immunohistochemical analysis (Figure 4). It has been previously reported that acivicin induces an increase of GSH concentration in the kidney through an unknown mechanism [47]. Since acivicin inhibits *γ*-GT and leads to glutathionuria, it might be assumed that a decrease of GSH, rather than an increase, could explain this effect. However, in light of our results, we can hypothesize that also acivicin is able to evoke the same effects of ADT (i.e., GSH increase and CysGly decrease) in plasma. Experiments carried out with the IPK model demonstrate that kidney GSH concentration is mainly dependent on plasma GSH, and its transport from plasma is inhibited by the anion transport inhibitor probenecid. Conversely, GSH concentration in the kidney is poorly influenced by *de novo* synthesis, as evidenced by the minimal influence of either BSO or acivicin (Figure 6). Plasma GSH can enter renal proximal tubular cells by several carriers occurring in the basolateral membrane, the most abundant of which is represented by the Oat family (Oat1/3). Among different cellular fates, GSH can undergo *γ*-GT degradation, generating its constituent amino acids by combination with the activity of a dipeptidase. This pathway is typical of the BBM side of the renal proximal tubular cells that is particularly rich in these enzymes [48] (Figure 7). We cannot exclude that besides the effect on *γ*-GT activity, ADT may exert its effect on the renal GSH inhibiting these transporters. This possibility deserves further investigation.

The fact that GSH in plasma can regulate kidney GSH, in particular in PT and DT cells, and that renal GSH can defend such cells from the harmful effects of oxidants or xenobiotics, raises some questions: (i) How the interorgan transport and cycle of GSH can be regulated? (ii) Is there a diet that can change plasma levels of GSH? (iii) Is there any drug that can change the plasma levels of GSH? The main source of GSH in plasma is the liver, from which it is continuously delivered at the rate of 16.4 nmoles/min/ml plasma [49]. It is debated if GSH from the diet is absorbed as it is. It has been reported that in rats administered with high concentrations of GSH (50 mg/kg), plasma GSH doubled after 120 minutes and that this result is due to intact GSH and not to the intake of its amino acid precursors [50]. However, different studies failed to demonstrate an intestinal absorption of GSH in humans [51, 52]. Moreover, even if some vegetables and meats can have a GSH content in the millimolar range, it has been calculated that the maximum intake of GSH in humans is around 150 mg/day [53]. This amount is far less the amount administered by gavage to the rats in the abovedescribed experiments and substantially lower if compared with the amount needed to increase plasma GSH. Drugs able to enhance the concentration of circulating glutathione are, therefore, of interest, and some are under evaluation [54–57]. However, drawbacks have been reported. For example, they induce a minimal and uncertain increase in GSH concentrations that is not stable as most of the drugs used are GSH precursors with a short half-life and/or low bioavailability. ADT can represent a good candidate for such a pharmacological application because it acts not as a GSH precursor, but rather as inhibitor of its own hydrolyzing enzyme. Furthermore, ADT may have a protective effect against direct or secondary pro-oxidant compounds that target the kidney and other organs, such as acetaminophen, cisplatin, ochratoxin, cephaloridine, gentamicine, and heavy metals [58–63].

It is to remark that, notwithstanding the statistical significance of our data and their agreement with previous results [12], the small number of replicates for each experiment can represent a limit of this work.

In conclusion, our data indicate that ADT is a specific and efficient GSH enhancer, the activity of which is based on the inhibition of the extracellular GSH degradation by *γ*-GT. The main effect of ADT was detected in the kidney where after treatment of rats , GSH concentrations almost doubled. We propose that ADT worths further investigation astherapeutic agentto specifically protect renal cells from the toxic effects of xenobiotics and oxidative stress.

## Figures and Tables

**Table 1:** Plasma thiols and disulfides in rats orally treated with ADT. Rats (*n* = 18) were orally administered 1 mg/kg (b.w.) saline (*n* = 9) or ADT (*n* = 9) once a day. After 1 week, 2 weeks, or 3 weeks, low molecular mass thiols, low molecular mass disulfides, and *S*-thiolated proteins were measured in plasma (*n* = 3 for each group). Data are expressed as *μ*M. ^∗∗^*p* < 0.01 vs. vehicle group; ^∗^*p* < 0.05 vs. vehicle group.

Thiol	1 week		2 weeks		3 weeks	
	Vehicle group	ADT group	Vehicle group	ADT group	Vehicle group	ADT group
GSH	9.41 ± 1.25	20.8 ± 1.47^∗∗^	10.7 ± 1.03	20.2 ± 0.95^∗∗^	9.48 ± 0.078	19.4 ± 1.18^∗∗^
GSSG	1.54 ± 0.32	1.50 ± 0.21	1.29 ± 0.33	1.10 ± 0.17	1.60 ± 0.28	1.03 ± 0.41
GSSP	0.80 ± 0.07	0.76 ± 0.05	0.82 ± 0.03	0.94 ± 0.06	0.75 ± 0.08	0.55 ± 0.07
CysGly	7.10 ± 0.92	3.24 ± 0.51^∗∗^	8.54 ± 0.35	2.94 ± 0.48^∗∗^	7.91 ± 0.70	2.53 ± 0.46^∗∗^
CySSGly	6.42 ± 0.27	5.33 ± 0.46	6.61 ± 0.51	4.17 ± 0.70^∗^	6.90 ± 0.19	4.56 ± 0.11^∗∗^
CyGlySSP	5.12 ± 0.81	2.77 ± 0.34^∗∗^	5.86 ± 0.44	2.59 ± 0.40^∗∗^	5.03 ± 0.84	2.88 ± 0.46^∗∗^
Cys	13.7 ± 3.2	14.1 ± 2.0	13.0 ± 1.1	16.8 ± 3.8	12.4 ± 4.0	16.2 ± 3.3
CySS	60.3 ± 8.6	55.1 ± 4.4	58.4 ± 8.6	55.3 ± 9.1	63.7 ± 5.2	58.0 ± 6.3
CySSP	66.4 ± 10.2	56.6 ± 7.0^∗^	62.0 ± 8.3	53.3 ± 4.2^∗∗^	65.8 ± 6.0	52.5 ± 6.4^∗∗^
Hcys	0.81 ± 0.03	0.85 ± 0.06	0.72 ± 0.03	0.51 ± 0.02	0.77 ± 0.05	0.64 ± 0.08
HcySS	2.14 ± 0.09	1.19 ± 0.12^∗∗^	2.14 ± 0.54	1.26 ± 0.06^∗^	2.29 ± 0.84	1.11 ± 0.38^∗^
HcySSP	3.56 ± 0.13	0.95 ± 0.02^∗∗^	3.06 ± 0.19	1.17 ± 0.02^∗∗^	4.12 ± 0.37	0.99 ± 0.26^∗∗^

Abbreviations: GSH: glutathione; GSSG: glutathione disulfide; GSSP: protein mixed disulfides with GSH; CysGly: cysteinylglycine; CySSGly: cysteinylglycine; CyGlySSP: protein mixed disulfides with CysGly; Cys: cysteine; CySS: cystine; CySSP: protein mixed disulfides with Cys; Hcys: homocysteine; HcySS: homocysteine, HcySSP: protein mixed disulfides with Hcys.

**Table 2:** Oxidative stress parameters in blood in rats orally treated with ADT. Rats (*n* = 18) were orally administered 1 mg/kg (b.w.) vehicle (saline) or ADT once a day for three weeks. At one-week interval biomarkers of oxidative stress were measured in blood (*n* = 3 for each group). PSSG values in RBC membranes are expressed as pmoles/mg Hb. ^∗∗^*p* < 0.01 vs. vehicle group.

Parameter	1 week		2 weeks		3 weeks	
	Vehicle group	ADT^∗^ group	Vehicle group	ADT group	Vehicle group	ADT group
Plasma PTI	0.369 ± 0.023	0.271 ± 0.017^∗∗^	0.397 ± 0.024	0.248 ± 0.060^∗∗^	0.347 ± 0.010	0.240 ± 0.038^∗∗^
RBCs GSH/GSSG	389 ± 21	435 ± 27^∗∗^	358 ± 14	430 ± 36^∗∗^	392 ± 23	405 ± 13^∗∗^
Membrane PSSG	10.8 ± 0.8	4.6 ± 0.74^∗∗^	9.3 ± 0.12	3.6 ± 0.59^∗∗^	14.3 ± 1.1	4.4 ± 0.2^∗∗^
Plasma MDA (nM)	54.6 ± 2.1	20.5 ± 0.8^∗∗^	64.7 ± 4.2	22.8 ± 1.4^∗∗^	51.3 ± 7.0	22.5 ± 1.2^∗∗^

Abbreviations: ADT: anethole dithiolethione; PTI: protein thiolation index; PSSG: *S*-thiolated proteins; MDA: malonyldialdehyde.

**Table 3:** Activity of *γ*-glutamyltranspeptidase in several organs of rats treated with ADT. Rats (*n* = 18) were orally administered 1 mg/kg (b.w.) vehicle (saline) or ADT once a day for three weeks. At one-week intervals, the activity of *γ*-glutamyltranspeptidase (*γ*-GT) was measured in organ homogenates (*n* = 3 for each group). Data are expressed as mU/mg protein. ^∗^*p* < 0.05 vs. vehicle group, ^∗∗^*p* < 0.01 vs. vehicle group.

Organ	1 week		2 weeks		3 weeks	
	Vehicle group	ADT group	Vehicle group	ADT group	Vehicle group	ADT group
Liver	1.42 ± 0.03^†^	0.692 ± 0.087^∗^	1.50 ± 0.11	0.523 ± 0.098^∗∗^	1.65 ± 0.078	0.413 ± 0.055^∗∗^
Kidney	2840 ± 195	1850 ± 109^∗∗^	3010 ± 224	1865 ± 86^∗∗^	2964 ± 340	1698 ± 101^∗∗^
Heart	2.20 ± 0.11	1.52 ± 0.07^∗∗^	2.00 ± 0.16	1.56 ± 0.13^∗∗^	1.89 ± 0.11	1.43 ± 0.02^∗∗^
Lung	8.25 ± 0.54	4.45 ± 0.50^∗∗^	7.86 ± 0.55	5.12 ± 0.34^∗∗^	7.97 ± 0.30	5.10 ± 0.26^∗∗^
Brain	2.29 ± 0.14	1.68 ± 0.21^∗∗^	2.12 ± 0.18	1.68 ± 0.14^∗∗^	2.06 ± 0.15	1.46 ± 0.27^∗∗^
Testis	2.03 ± 0.13	2.41 ± 0.18	1.86 ± 0.07	2.24 ± 0.20	2.67 ± 0.35	1.37 ± 0.09^∗∗^

Abbreviations: ADT: anethole dithiolethione.

**Table 4:** GSH metabolism in isolated perfused rat kidney treated with glutathione alone or in association with acivicin or probenecid. Isolated rat kidneys were perfused for 2 hours with 10 *μ*M GSH (*n* = 3) 20 *μ*M GSH (*n* = 3), 20 *μΜ*GSH  + 0.1 mM probenecid (*n* = 3), 20 *μΜ*GSH  + 0.5 mM acivicin (*n* = 3). Urine was collected by cannulating ureter. Samples were collected at 30, 60, and 90 minutes from the beginning of the perfusion from the reservoir connected to the renal artery and from the renal vein outflow for GSH analyses and calculation of its extraction rate. Urinary tGSH values are expressed as nmoles/mg creatinine, ^∗∗^*p* < 0.01 vs. GSH 10 *μ*M group.

Parameters	GSH 10 *μ*M	GSH 20 *μ*M	GSH + acivicin	GSH + probenecid
Extraction % (30′)	72 ± 5.0	70.6 ± 3.7	75.8 ± 8.8	47.5 ± 7.6^∗∗^
Urinary tGSH	0.18 ± 0.02	0.20 ± 0.05	105 ± 21^∗∗^	0.19 ± 0.01

Abbreviations: GSH: glutathione; tGSH: total glutathione.

## Data Availability

The data used to support the findings of this study are available from the corresponding author upon request.
